# Cancer quasispecies and stem-like adaptive aneuploidy

**DOI:** 10.12688/f1000research.2-268.v1

**Published:** 2013-12-06

**Authors:** Domenico Napoletani, Michele Signore, Daniele C Struppa

**Affiliations:** 1 Institute for Quantum Studies, Chapman University, Orange, CA, 92866, USA; 2 Department of Hematology, Oncology and Molecular Medicine, Tumor Stem Cell Biobank, Istituto Superiore di Sanita, 00161, Rome, Italy; 3 Schmid College of Science and Technology, Chapman University Chapman University, Orange, CA, 92866, USA

## Abstract

In this paper we develop a theoretical frame to understand self-regulation of aneuploidy rate in cancer and stem cells. This is accomplished building upon quasispecies theory, by leaving its formal mathematical structure intact, but by drastically changing the meaning of its objects. In particular, we propose a novel definition of chromosomal master sequence, as a sequence of physically distinct whole or fragmented chromosomes, whose length is taken to be the sum of the copy numbers of each whole or fragmented chromosome. This fundamental change in the functional objects of quasispecies theory allows us to show that previously measured aneuploidy rates in cancer populations are already close to a formally derived aneuploid error threshold, and that any value of aneuploidy rate larger than the aneuploid error threshold would lead to a loss of fitness of a tumor population. Finally, we make a phenomenological analysis of existing experimental evidence to argue that single clone cancer cells, derived from an aneuploid cancer subpopulation, are capable of self-regulating their aneuploidy rate and of adapting it to distinct environments, namely primary and metastatic microenvironments. We also discuss the potential origin of this self-regulatory ability in the wider context of developmental and comparative biology and we hypothesize the existence of a diversification factor, i.e. a cellular mechanism that regulates adaptation of aneuploidy rates, active in all embryo, adult and cancer stem cells.

## Introduction

In normal cells the number of chromosomes and the total DNA content depends on the phase of the cell cycle ^1^. Non-diploid chromosome content, also known as aneuploidy, is instead the most common feature of human tumor cells ^2– 4^. This feature of tumor cells is commonly associated with acquired resistance to various kinds of treatments such as radio- or chemotherapy ^2– 5^. Nonetheless, it is not completely clear whether aneuploidy per se contributes to and drives tumor development, or if instead it is deleterious. In fact, individuals carrying an extra copy of chromosome 21 have a 50% lower probability of developing solid tumors than do individuals with the correct chromosome number ^6, 7^ and although aneuploidy is compatible with organism and cell viability, the presence of additional copies of chromosomes decreases the overall cellular fitness ^8^. What is clear is that chromosomal instability, i.e. the tendency to gain or lose parts of the genome during cell replication, seems to give an advantage to tumor cells ^9^, and clonal heterogeneity within tumors is one of the main causes of tumor dormancy and resistance to anti-cancer therapies ^5, 10^. Indeed, it has been demonstrated that even a small population of cells derived from a single cancer cell clone (diversified population from a single cell), could be responsible for drug resistance and, upon removal of the drug, the population spontaneously reverted to a sensitive state ^11, 12^.

The development of single-cell sequencing techniques ^13^ has recently allowed a wide range of studies analyzing chromosomal variability in primary and metastatic tumor cells, as well as healthy tissues ^14, 15^. In particular in Navin *et al.* ^16^ it is shown that metastatic tumors are likely to be the product of single clones proliferating from the primary tumor by observing the microvariation of the integer copy number of consensus sequences in individual tumor cells through single-cell sequencing.

In this paper we are concerned with measurable changes in the aneuploid heterogeneity of populations, due to different levels of chromosomal instability, and to emphasize even more the close link between aneuploidy and chromosomal instability. We will refer to the latter as *aneuploidy rate*, formally defining aneuploidy rate as the average probability that there is at least one new aneuploid modification per chromosome during cell replication. We will demonstrate in the Discussion that simple and verifiable statistical consequences of the findings of Navin *et al.* ^16^ are logically bound to imply the following proposition.

**Adaptive Aneuploidy in Cancer Cells:** *Single clone cancer cells derived from an aneuploid cancer subpopulation are capable of adapting their aneuploidy rate and display distinct aneuploid rates in distinct environments, namely primary and metastatic microenvironments*.

The full impact of this proposition hinges on the potential ability to harness adaptive aneuploidy and potentially alter the aneuploidy rate of cancer cells. The theoretical frame for such a program can be built on quasispecies theory, a general evolutionary model for error-prone self replicative systems ^17^, first introduced by Eigen ^18^. The potential relevance of quasispecies theory to cancer biology has already been suggested ^19, 20^ but unlike previous attempts ^20– 26^, we do not recommend in this paper alternative, structurally diffnt quasispecies models of cancer cell subpopulations dynamics. Instead, after a quick review of the relevant elements of standard quasispecies theory, we note the limits of this theory for the study of eucaryotic cells, and show that only by defining a novel notion of chromosomal master sequence it is possible to radically shift the whole standard quasispecies theory to a new categorical context (i.e. the scenario of cell populations with variable aneuploidy rates) and properly justify a valid *aneuploid quasispecies theory*. This new context allows us to predict (similarly to what the standard quasispecies theory does for single base error rates) a maximal aneuploidy rate, an error threshold, after which each cancer subpopulation loses its identity, and therefore its ability to pass on its selectively advantageous genetic traits to future cell generations, which is referred to as error catastrophe ^27^.

Finally, in the Discussion we perform a phenomenological analysis of some especially illuminating single cell analysis experiments, from Navin *et al.* ^16^, that seem to support our proposition on the existence of adaptive aneuploidy in cancer cells. We then speculate on the possible biological basis of self-regulation of aneuploidy rates in cancer cells. Such refined ability is unlikely to be the product of a single, specific tumor evolutionary history, rather, we will argue that embryo and adult stem cells already display very finely regulated rates of aneuploidy, suggesting that a common cellular mechanism of adaptation of aneuploidy is at play, a *diversification factor*, which is possibly reactivated in cancer stem cells.

## Aneuploid quasispecies

### The quasispecies framework

As noted in the Introduction, we need to redefine the main quantities at play in quasispecies theory to make it an appropriate ground for the biology of aneuploidy, and we begin by briefly sketching the argument that leads to the error threshold inequality.

The concept of quasispecies was first introduced by Eigen ^18, 28^, and it is a powerful way to relate the structure of population dynamics to the error rate of single base replication in viruses or unicellular organisms ^27, 29^. The most important consequence of the theory is that it is possible to determine theoretically a threshold on the error rate such that, if the error rate of replication of genomic sequences is pushed above the threshold, the subpopulation will not be able to retain its identity, for a wide range of models for fitness distribution in the population (cf. Schuster ^30^, page 81).

Assuming there are *N* subpopulation types within a population, we start by writing down the differential equation that describes the rate of change *dx _m_* of type *m* in terms of the instantaneous size *x _k_*( *t*), *k* = 1, ..., *N* of all types:

$$\frac{dx_{m}}{dt}=(W_{mm}-\bar{E}(t))x_{m}(t)+\sum_{k\neq m}W_{mk}x_{k}(t),\;\;\;\;\;(1)$$

*W _mm_* is the rate of effective excess production of sub-population type *m*, and if we consider the genetic sequence associated to type *m*, we can write *W _mm_* = *Q _mm_A _m_ − D _m_*, with *Q _mm_* the probability of precise reproduction of sequence *m*, *A _m_* the growth rate of type *m*, and *D _m_* its mortality rate. *Ē*( *t*) = Σ *_k_ E _k_ x _k_* is the average, over all types, of the excess reproduction rate, with *E _k_* = *A _k_ − D _k_* and, finally, *W _mk_* is the rate of production of type *m* by erroneous reproduction of type *k*.

Assuming a steady state in which *dx _m_/dt* = 0 and neglecting in first approximation the contributions Σ *_k≠m_ W _mk_ x _k_*( *t*), it is possible to derive a condition that constrains the probability of precise reproduction of sequence *m*:

$${\bar{\sigma }}_{m}Q_{mm}>1,\;\;\;\;\;(2)$$

where *$\bar{\sigma }$_m_* = *A _m_/*( *D _m_* + *Ē _k≠m_*), with *Ē _k≠m_* = Σ *_k≠m_ E _k_ x _k_*, is the average superiority of a master sequence associated to a dominant subpopulation versus competitor sequences, essentially, *$\bar{\sigma }$_m_* is an index of relative fitness ^27^. If a master sequence has length *v _m_*, and we denote by *$\bar{q}$* the average fidelity of single nucleotide reproduction, then *Q _mm_* = *$\bar{q}$^v _m_^* and the error threshold can be written as

$$\frac{l\text{n}{\bar{\sigma }}_{m}}{v_{m}}\geq 1-\bar{q}.\;\;\;\;\;(3)$$

Remarkably, Equation 3 establishes a phase transition on the information content; if the error rate of single nucleotide reproduction goes above $\frac{l\text{n}{\bar{\sigma }}_{m}}{v_{m}}$ the information contained in the master sequence will disintegrate, in the sense that the loss of information in the sequence due to reproduction errors will not be compensated by a sufficiently high fitness relative to other subpopulations and the subpopulation associated to the master sequence will implode ^27, 30^.

### Limitations of quasispecies theory in eucaryotic cells

In complex organisms, the quasispecies model is potentially applicable only in specific scenarios, such as competition among embryo stem cells during development, adult stem cells and progenitor cells proliferation, and, crucially, cancer cells, where subpopulations compete with each other under limited resources and changing environment. However, the applicability of the basic quasispecies model, originally devised in the setting of virus RNA replication, has been put into question as appropriate for eucaryotes and specifically for cancer cells. Eucaryotic cells reproduce semi-conservatively meaning that the parental double strand degenerates in the process of generating two daughter double strands, which led to Brumer *et al.* ^21^ to raise the possibility that, for high enough replication error rates, the master sequence, seen here as the double strand of DNA, would eventually disappear, and they suggested more refined quasispecies models that take into consideration this phenomenon.

Even more seriously, the applicability of quasispecies theory to human cells is put into question by the exceedingly high size of the human genome as compared to RNA viruses. In fact, in order for the quasispecies not to undergo genetic drift, the neutral space around a fitness peak should be sufficiently small to be completely explored by the population. The complexity and the inherent mutational and phenotypical robustness of the human genome amplifies its neutral space, preventing quasispecies evolution even at higher than normal mutation rates, as it is the case in cancer cells ^31^. This fact, together with very low single nucleotide errors for humans, implies that the fitness of mutants of an hypothetical master sequence does not change significantly, and the fitness distribution of mutants around the master sequence is likely to decay linearly, or sub-linearly, a scenario under which no error threshold is possible ^30^.

Indeed, the existence of Lynch syndrome or hereditary non-polyposis colorectal carcinomas (HNPCC), which are characterized by a higher risk of colon cancer, show the inability of the basic quasispecies theory to predict the maximum single nucleotide error that is viable for a tumor. HNPCC tumors, as well as all microsatellite instable (MSI) colon cancers, arise because of a break down of the mismatch repair mechanism ^32, 33^. Therefore, MSI cancer cells display increased error rates of single nucleotide replication by 1 to 3 orders of magnitude, with respect to the baseline single nucleotide error rate 1 − *$\bar{q}$* in healthy cells, estimated to range between 10 ^−9^ and 10 ^−10^ for the human genome (see Alberts *et al.* ^34^, page 271, and Gundry & Vijg ^35^, Lange *et al.* ^36^ and Jiricny ^37^).

Now recall that the human genome has roughly 3.2 × 10 ^9^ nucleotides (see Alberts *et al.* ^34^, page 206), and note that for organisms with very large genomes, the relative superiority *$\bar{\sigma }$_m_* of a master sequence associated to a given subpopulation cannot be very large (vis a vis other subpopulations), as any given mutation will only affect its fitness marginally ^27^, and therefore *$\bar{\sigma }$_m_* ≈ 1. Given these numerical estimates, according to the error threshold inequality in Equation 3, MSI tumors would fail to satisfy the error threshold inequality to such an extent that they should not even exist. This is true even if we restrict our attention, in defining the master sequence, to conserved DNA, i.e. the 5% of the human genome that is known to be coding and essential to cell function (see again Alberts *et al.* ^34^, page 206).

### Chromosomal master sequences and aneuploid error thresholds

We believe that the inconsistences of the basic quasispecies model, when applied to human cells, completely disappear if we replace single nucleotide errors with aneuploidy errors. Notably, in all scenarios where quasispecies theory could potentially apply, i.e. stem and progenitor cells proliferation and cancer cells, aneuploidy rates far exceed single nucleotide error rates in frequency and impact on the cell. This means the leading cause in the evolution of a population will be the aneuploidy error, rather than the single nucleotide error, which can be neglected, especially when the mismatch repair genes are intact as happens in the overwhelming majority of cancers and all healthy stem cells.

We need now to reinterpret the notion of reproduction fidelity of a sequence adequately to properly define error thresholds in the presence of aneuploidy. Since we can neglect nucleotide errors, we assume a faithful reproduction of the genetic material when the copy number of each chromosome in a sequence (two, for example, in a diploid cell) is kept constant during replication, both numerically (number of physically distinct whole chromosomes or fragments) and structurally (translocations, deletions and amplifications of DNA). Although complex aneuploidy landscapes may arise, characterized by concurrent numerical and structural chromosomal changes, most of the somatic copy-number alterations (SCNAs) frequently found in tumor cells involve whole chromosomes or whole-arms (25% of the genome), with only 10% of the cancer cell genome being affected by focal SCNAs ^9, 38, 39^.

Therefore, we now make a series of definitions consistent with these arguments, whose objective is to change the domain of applicability of quasispecies theory, without altering its formal mathematical structure:

**Chromosomal Master Sequence.** *A chromosomal master sequence is the collection of physically distinct whole and fragmented chromosomes in the cell*.

**Chromosomal Master Sequence Length.** *The chromosomal master sequence length c _m_ of a cell is the sum of the copy numbers of each whole or fragmented chromosome in its nucleus*.

**Aneuploid Fidelity.** *The aneuploid fidelity *Ā _m_* is the average probability that each whole or fragmented chromosome is reproduced exactly once in cell division, with no gain or loss of sub-chromosomal regions*.

In this aneuploid scenario, the chromosomal master sequence length *c _m_* can fluctuate depending on the number of aneuploid copies of whole chromosomes or fragments, and the underlying nucleotide sequence will clearly differ according to which chromosomes or individual genes are affected by copy-number alterations in each cell. Although tumors vary widely in the number and type of copy number changes, most of these comprise low-level alterations and only a few genes reach more than 20 copy numbers, mainly due to their oncogenic or drugresistance functions ^16, 40– 42^.

Aneuploid events can cause large phenotypical variations ^43– 46^, even a single error leading to chromosomal loss or addition can have large effects, therefore the fitness distribution around a master sequence is expected to display a sharp decay from the master sequence peak, in line with the types of fitness distribution known to express the error threshold ^30^. At least for cancer cells, sub-populations are sharply defined in terms of their aneuploid profile, as evinced from single cell analysis works ^16^ commented in the Discussion. This is further evidence of the strong concentration of fitness distributions around a few chromosomal sequence types.

Mutants of the master sequence, generated by even a single aneuploid error, and individual cells belonging to other sub-populations, are exceedingly unlikely to be able to mutate into cells expressing the master sequence, since any additional (erroneous) chromosome copy is subject to a wide variety of further partial deletions/additions, and only very few of them would correspond to a return to the master sequence configuration. Essentially, we can assume that the contribution of cells belonging to other subpopulation types to the dynamical evolution of the master sequence subpopulation is very small. This is exactly the condition that led to the error threshold in the first place, since Equation 3 is derived as a limiting stationary behavior of an interacting family of subpopulations described by Equation 1, where the rate of growth of each of them is weakly affected by the cross-mutations derived from the other subpopulations ^27, 30^.

Given these caveats regarding distribution of fitness for chromosomal master sequences in the presence of aneuploidy and regarding sub-population interactions, we reach the conclusion that quasispecies theory is indeed applicable to cancer and stem cells, but only in the context of aneuploid chromosomal master sequences, neglecting the underlying nucleotide errors.

We can now replace variables in the error threshold inequality in Equation 3 to take into account not only the varivariable length *c _m_* of the chromosomal master sequence associated to all whole and fragmented chromosomes, but also the correction to the probability *Q _mm_* of precise reproduction of a sequence that aneuploidy entails. The probability of precise reproduction of a specific sequence of chromosomes *m* can be expressed as *Q _mm_* = *Ā ^c _m_^_m_*, and the aneuploid error threshold inequality can be written as *$\bar{\sigma }$_m_Ā ^c _m_^_m_* > 1, which eventually gives us a standard form (formally identical to Equation 3) for the **aneuploid error threshold inequality:**

$$\frac{l\text{n}{\bar{\sigma }}_{m}}{c_{m}}\geq 1-{\bar{A}}_{m},\;\;\;\;\;(4)$$

However each term in this equation has drastically different orders of magnitudes than the threshold inequality for DNA or RNA master sequences. To start with, as already stressed above, the relative superiority *$\bar{\sigma }$_m_* will have considerable fluctuations, since the chromosomal master sequence is much shorter than a nucleotide sequence, and even small variations in copy numbers can effect large phenotypical variations.

At the same time, the diversity of subpopulations in primary tumors ^16, 47– 51^ implies that, in a fully developed tumor, different subpopulations do not have extremely different relative superiority *$\bar{\sigma }$_m_*, a scenario that would lead to a single, highly dominant subpopulation. It is therefore reasonable to assume at the very most *$\bar{\sigma }$_m_* ∈ [10 ^2^, 10 ^3^], for the subpopulations of highest relative superiority. We know moreover that highly aneuploid tumors have higher fitness ^52^, so larger values of *$\bar{\sigma }$_m_* are likely to be associated with large values of *c _m_*, up to the order of 10 ^2^ ^13, 39, 53, 54^.

Let *E _m_* = 1 – *Ā _m_*, with *E _m_* denoting the aneuploidy error rate, i.e. the average probability that there is at least one new aneuploid defect for each chromosome or fragment of chromosome during cell replication. If we call *T*( *E _m_*) the threshold aneuploidy error rate above which a chromosomal master sequence is not viable, and if we take *$\bar{\sigma }$_m_* ∈ [10 ^2^, 10 ^3^], *c _m_* ≈ 10 ^2^, then Equation 4 gives *T*( *E _m_*) ≈ 10 ^–2^, which is consistent with the estimates of *E _m_* for cancer cells, in the range [10 ^–3^, 10 ^–1^] ^3, 55– 58^. Our argument implies that the more a cell is aneuploid, the tighter the error threshold bound is, and that highly aneuploid cancer cells, known to be most adaptable ^5, 52, 59, 60^, are already working with aneuploid error rates close to the limit of a viable quasispecies.

There is some evidence that indeed aneuploidy rates in the tumor can affect the prognosis of cancer patients ^9, 61^. It is suggested that a moderate tumor aneuploidy rate worsens the prognosis, while a very high aneuploidy rate is associated with improved patient outcomes ^9^, consistent with the quasispecies and error threshold catastrophe approach.

## Discussion

We would like to revisit and comment upon some specific experimental evidence for our proposal of a self-regulated aneuploidy rate in cancer cells, focusing on several measures of aneuploidy rate, and showing how the variability observed in tumor subpopulations subject to distinct micro-environments can be given a far-reaching interpretation. Our analysis is phenomenological, meaning that we explain and reinterpret existing experimental work, showing how the inner logic of our argument can severely constraint the causes and interpretation of the data we review. We chose to first perform an in-depth analysis of a single recent study ^16^, so that the flow of our discourse is unified and made coherent by constant reference to the same context. At the same time, we support our arguments with related experimental works, when appropriate.

The in-depth analysis and commentary of the literature that we perform here is meant to show that there is rigorous, logically compelling, and experimentally testable biological evidence for the usefulness of an aneuploid quasispecies theory. Indeed, we argue that it is possible to envision a precise, and functionally in-built mechanism of self-regulation for aneuploidy rates in normal and cancer stem cells. The evolutionary role and the structure of this self-regulation mechanism could be studied and conceptualized within the framework we developed for aneuploid quasispecies. At the same time, any experimental validation of aneuploid quasispecies predictions on threshold aneuploidy error rates, would be essentially related to the presence of this self-regulatory mechanism.

### Evidence for self-regulation of aneuploidy rates in cancer cells

The main focus and objective of Navin *et al.* ^16^ was to show that metastatic tumors are likely to be the product of single clones proliferating from the primary tumor by observing the microvariation of the integer copy number of consensus sequences in individual tumor cells through single-cell sequencing. A coarse ploidy distribution of a large number of cells from a breast tumor and one of its metastasis was plotted in Navin *et al.* ^16^ (Figure 3a-b in that paper) as an histogram with respect to the total DNA content. These ploidy distributions showed, for both primary and metastatic tumors, two peaks, one around twice and another at four times the total amount of DNA. This double peaked distribution is accounted by the presence of roughly 50% of normal diploid cells in each tumor tissue sample. Importantly, whereas in the primary tumor a significant fraction of the gated normal cells was pseudodiploid, in the paired metastasis the normal population was likely to derive from the stromal content of the tissue (see ref. ^16^, Figure 4 in their paper).

Navin *et al.* ^16^ performed very refined measurements of copy number profiles, across all chromosomes, from a small subset of cells (hundreds) in sections of primary and metastatic tumors and generated a neighbor-joining tree of these profiles. This analysis showed that metastatic and primary aneuploid cells were closely related in the neighbor-joining tree derived from the clustering, and yet they produced clearly distinct subclusters. The conclusion of this single cell study was that the metastasis proliferated from a single cell derived from the aneuploid subpopulation of the primary tumor, since no pseudodiploid cancer cells were observed in the metastatic tumor.

Other recent studies on myeloproliferative disorders ^62^ and melanoma ^54^, kidney ^47, 48^ and pancreas ^49^ tumors or, again, in breast cancer ^50^, arrived at similar conclusions, pointing to a late, metastasis-specific diversification of primary tumor-derived cells (see Wu *et al.* ^63^, Figure S14, and Clifford ^64^). The presence of aneuploid cells in most primary tumors examined in Gerlinger *et al.* ^49^, is well documented by ploidy analysis (see supplementary Figure 10 in their paper) and metastases show a marked increase in allelic imbalance as compared to primary tumor regions. The authors conclude that tumor heterogeneity is probably driven by aneuploidy and that chromosomal aberrations contribute substantially to genetic intratumor heterogeneity. Notably, even in an evolutionary context where the primary tumor and the metastasis share most of the sequenced regions, there is a striking variation in copy number specifically in the metastatic counterpart (see ref. ^54^, Figures S5 to S11 in Lengauer *et al.* ^54^). This concept is best exemplified in ref. ^16^, but there, both the coarse ploidy distribution analysis and the refined, single cell copy number count for primary and metastatic tumors, drive us to an additional conclusion: *the genetic variability of the aneuploid clone in the metastasis is greater than its corresponding variability in the primary tumor from which it came*. Even if we took into account a parallel progression model as opposed to a punctuated or linear evolution model ^51, 65^, the final result would not change. The metastatic population described in Navin *et al.* ^16^ has diversified more than its parental population in the primary tumor, regardless of whether the metastasis developed in a later, much shorter time than the primary tumor, or whether its origins date back to the first stages of primary tumor dissemination. This higher diversification of the metastatic population holds even though the aneuploid cells’ compartment in the primary tumor does not represent a minority of the population, and has expanded considerably at some point during the tumor evolution history.

To justify our claim of adaptive aneuploidy, we note that in Figure 4 of Navin *et al.* ^16^ the Euclidean distances in the neighbor-joining tree for the aneuploid cells from the metastatic tumor showed much greater variability than the Euclidean distances for the corresponding aneuploid cells in the primary tumor. We note that these distances were calculated with respect to a common root profile, and that mutual distances among individual profiles are likely to be ever greater. Granted that this study dealt with very small sample populations, a closer inspection and analysis of the tightness of the variance of Euclidean distances in the primary tumor subpopulation, as opposed to the variance of Euclidean distances of the metastatic tumor subpopulation, would almost certainly reveal a statistically significant discrimination of the two. Indeed, distances inferred from their Figure 4 of ref. ^16^ using a Levene test for equality of variance ^66^ suggest a low probability that the underlying distributions of Euclidean distances for metastatic aneuploid cells and for primary aneuploid cells have the same variance (p-value ≈ 0.01).

Note, crucially, that even if we assume high experimental noise in the data, such noise would affect equally *both* Euclidean distributions and the identification of distinct variances for distances of metastatic aneuploidy cells and primary aneuploidy cells would be even more unlikely to be observed by chance.

The hypothesis of a larger variability of the copy number profiles of the metastatic subpopulation is also supported by a closer inspection of the tails of the ploidy distributions of primary and metastatic tumor populations analyzed in Navin *et al.* ^16^. The right-hand sides of the tetraploid peaks for the metastatic tumor have distinctly thicker and longer tails than the corresponding tetraploid peaks in the primary tumor, suggesting greater variability of aneuploidy in the former. Other works point to a similar conclusion. For example, it has been shown recently that, although sharing most of the examined somatic single-nucleotide variants, *in vitro* cultured low passage melanoma cells have higher copy number variation when compared to the parental tumor ^67^.

Ploidy distributions, in their simplicity, offer even more scope for interpretation and testing of the hypothesis that cancer cells have the ability to self-regulate their aneuploidy rate. Indeed, a single cell clone could be capable of generating a diverse metastasis either because of inherent chromosomal instability, or because its rate of aneuploidy is somehow increased under the stress conditions of a new tissue embedding.

Let’s assume first that the single clone from the primary tumor has chromosomal instability. The dispersion of metastatic cells should not be in any way preferential to such cells (even if their successful embedding in a tissue may be), so there will be a, possibly small, subpopulation of cells in the primary tumor with similar or higher aneuploidy rate than the cell generating the metastasis. This subpopulation of the primary tumor, by its greater aneuploidy rate, will be more adaptable and likely self-sustaining, and it should be observable as a long tail in the ploidy distribution of the primary tumor population. The tail will be much thinner for the primary tumor than the metastatic tumor, since high aneuploidy rate cells are only a sub-population of the primary tumor. However, *no such long and thin tail is observed experimentally for the primary tumor* in Navin *et al.* ^16^. It is still possible, if highly unlikely, that the metastatic cell is an extreme outlier, with no comparable cells left in the primary tumor, but then we would see a much more pronounced evolutionary difference between primary and metastatic aneuploid populations than what is observed. This logically implies that the single metastatic cell clone was not essentially different from its primary population before starting to proliferate in the new environment.

This argument leaves only one other option: some cancer cells are capable of altering and self-regulating their aneuploidy rate under stress, or under changes in the environment. In conclusion, both ploidy distribution analysis and single cell analysis of Navin *et al.* ^16^ give strong evidence for the proposition on adaptive aneuploidy in cancer cells presented in the Introduction, i.e. that *single clone cancer cells derived from an aneuploid cancer subpopulation, adapt their aneuploid rates in distinct environments, namely primary and metastatic microenvironments*.

### A diversification factor

The Discussion, up to now, centered on the inference, from some experimental results of ref. ^16^ and other published supportive information ^48, 54, 63^, that metastatic cancer cells have higher aneuploidy rates than the corresponding original subpopulation of the primary tumor. We concluded that this differential could only be explained by assuming an adaptive, self-regulatory cellular response sensitive to changes in the environment. As much as tumor populations undergo extensive evolution during their development, it seems highly unlikely that such a refined property could arise by chance only in the specific population studied in Navin *et al.* ^16^, without a preexisting dormant ability to self-regulation.

Indeed, high levels of aneuploidy are associated with increased adaptability in plants and yeast ^68, 69^, and a certain rate of aneuploidy, leading to precise percentages of mosaic aneuploidy, is common in several mammals’ embryos (see van Soom & Boerjan ^70^ chapter 10), including humans ^71, 72^. Similarly, it is speculated that the significant mosaic aneuploidy in adult human organs such as the liver and brain is instrumental to an increased plasticity and adaptability of such organs ^73, 74^. Lang *et al.* ^75^ raise the possibility that the extensive aneuploidy in the embryo may transfer into similarly widespread copy number variations in all human tissues.

Observable levels of aneuploidy have been found in adult cells ^76^, and while this widespread aneuploidy could already originate during embryo development ^75^, adult, non-transformed stem cells continue to have distinct levels of aneuploidy rates according to their type. For example, mesenchymal stem cells are likely to have very low aneuploidy rates ^77^, while hepatocytes together with small intestine and pancreas cells display within-tissue extensive copy number variation (CNV) ^78, 79^.

These strikingly different aneuploidy rates among embryo stem cells and adult stem cells, raise the possibility that the finely tuned, and distinct, high aneuploidy rates observed in embryos and adult tissues are regulated by some mechanism specific to stem cells, rather than being a simple byproduct of aberrant or sustained cell division. This is a simple, fundamental observation, and yet one that is rarely emphasized in the literature. We formalize its essence in a proposition:

**Differentially Expressed Aneuploidy in Stem Cells.** *Embryo and adult stem cells display finely tuned, and distinct, aneuploidy rates, unrelated to the replication rates of the stem cells themselves*.

And we refer to Section 2 of our preprint ‘Stem-like Adaptive Aneuploidy and Cancer Quasispecies’ (available at arxiv.org/pdf/1303.6374.pdf) for a much more thorough review of literature supporting the idea of a differentially expressed aneuploidy in stem cells.

Since many types of cancers partially inherit the hierarchical structure of the tissues they have derived from and are assumed to be propagated by stemlike tumor cells ^80^, it is possible that increased aneuploidy rates are used actively, to the population advantage, to increase the adaptability of stem or fast-dividing progenitor cells. It is worth noting that, to date and to our knowledge, only two studies assessed the relationship between aneuploidy and cancer stem cells (CSCs) ^81, 82^. In the first paper, Kusumbe and Bapat evaluated the expression of stem-cell markers and the DNA content distribution of fluorescently labeled ovarian cancer cells after subcutaneous injection into immunodeficient mice. The authors found that, unlike label-free tumor cells, the label-retaining (quiescent) cells displayed stem-cell markers and were embedded with a small fraction of aneuploid cells. Treatment with chemotherapy increased the percentage of quiescent cells in the overall population and selectively stimulated the proliferation of the aneuploid fraction, which retained stem-like properties upon removal of the drug ^81^. A second study by Fujimori *et al.*, reveals again that stressful conditions favor the emergence of CSC-like clones from differentiating embryo stem cells in *in vitro* culture ^82^.

And this brings us to the proposition we stated in the introduction on adaptive aneuploidy in cancer cells: in light of the refined use of aneuploidy in stem cells, self-regulation of aneuploidy as argued in this Discussion could be a reactivation, *in cancer stem cells*, of a preexisting cellular mechanism common to embryo and adult stem cells - a diversification factor. Note that the proposition on differentially expressed levels of aneuploidy in normal stem cells is not compatible with a random, self-catalytic increment of aneuploidy first suggested in Rasnick & Duesby ^45^, since otherwise we would not be able to observe consistently similar levels of aneuploidy within each stem cell type, and among different individuals. Moreover, we would see a correlation between fast replicating stem cells and levels of aneuploidy in the corresponding tissue. This is not necessarily the case. For example the percentage of cells undergoing DNA replication in solid tumors, which are mostly aneuploid, varies between 2% to 8%, whereas a normal renewing epithelium such as the intestine exhibits a DNA replication index of approximately 16% ^83^.

A random progression of aneuploidy would display much more pronounced variability within each stem cell class, while the spread of aneuploidy would be similar in all stem cell classes, and not differentially expressed in each of them. Note that the theory of a self-catalytic origin of aneuploidy in principle could be made consistent with the proposition on adaptive aneuploidy in cancer. It is only by taking together the proposition on adaptive aneuploidy in cancer (stem) cells, and the proposition on differentially expressed aneuploidy in (all) stem cells that we argue the following hypothesis is strongly implied:

**A Diversification Factor:** *There exists a cellular mechanism that regulates adaptation of aneuploidy rates, active, to different degrees and in different modalities, in all embryo, adult and cancer stem cells*.

## Conclusions

While it was not the main objective of this theoretical paper to explore the experimental consequences of our hypothesis on adaptive aneuploidy and aneuploid quasispecies, we note that all our statements are open to, indeed they invite, simple forms of validation, either through single cell analysis or ploidy distribution analysis. Elucidating the mechanisms underlying self-regulation of aneuploidy rates in stem cells under specific microenvironmental stresses, would provide crucial insight into the developmental and evolutionary processes of complex organisms.

Finally, we emphasize that validation of the hypothesis that stem cells can adapt their aneuploidy rate through a diversification factor would have significant therapeutical implications, when specialized to cancer stem cells. Indeed, it would provide a biological way, mostly inactive or less sensitive in healthy adult cells, to induce an aneuploid quasispecies error catastrophe to weaken cancer populations, a long held hope that may yet prove itself true.
