# Supplementary material for: Cancer quasispecies and stem-like adaptive aneuploidy
Source: F1000Res. 2013 Dec 6;2:268. [Version 1] doi: 10.12688/f1000research.2-268.v1 (PMC3968898; doi:10.12688/f1000research.2-268.v1)
Supplement: Peer review history

# Peer review: Referee response for version 1

**Published:** 2014-03-10

**Authors:** Samuel F Bakhoum

**Affiliations:** 1 Mount Auburn Hospital, Harvard Medical School, Boston, MA, USA

In this interesting opinion article, Napoletani *et al.* attempt to apply the quasispecies theory to the evolution of anueploidy in cancer. This article proposes some interesting points that attempt to address the origin of genetic heterogeneity in tumors. An important claim made therein is that aneuploidy is likely to be the major source of genetic heterogeneity in later stages of tumor development particularly when compared to the rate of individual DNA mutations. Further, the authors also posit that cancer cells must maintain a given rate of chromosome missegregation beyond which a meltdown occurs.

I think this article is acceptable, but feel the authors should address the following points:

- Please change the "aneuploidy rate" into "chromosome mis-segregation rate" or something of that nature. Aneuploidy by definition is a state and not a rate. What the authors imply is "the rate of change in aneuploid karyotypes which is synonymous to chromosome mis-segregation (which includes whole chromosome missegregation as well as partial i.e. translocation etc...)
   
- It is important throughout the article to make the distinction between tolerance for aneuploidy in normal vs. cancer tissues. While evidence suggests that aneuploidy may lead to cellular stress in normal tissues, it is unclear whether this is the case in cancer cells. Either cancer cells preferentially maintain aneuploidy at the expense of increased cellular stress and slower proliferation rate or they might instead have also evolved to overcome cellular stress in response to aneuploidy. Furthermore, constitutional aneuploidy (for instance in individuals with trisomy) may have gone through a stem-cell adaptation phase that is different when compared to cancer cells who acquire aneuploidy de novo.
   
- The concept of error catastrophe is very interesting and rather important in the field of aneuploidy and chromosome segregation. I would encourage the authors to further elaborate on the optimal T(Em) value being near 10 ^-2^. Can the authors use the quasispecies model to make predictions for the fitness of the tumor cell populations if the T(Em) values were much higher? This would be extremely valuable.
   
- In addition to chromosome segregation errors (which include whole and partial chromosome copy number changes) it would be useful to incorporate the newly uncovered massive rearrangements known as chromothripsis and chromoplexy. In some cases, these massive rearrangements can lead to double minute chromosomes carrying oncogenes that are present in hundreds of copies (a lot higher than the maximum rate of 20 copies for any given gene that the authors presume). The authors do not account for these possibilities in their model.
   
- A concept that the authors bring up is the presence of a feedback loop from the environment that can "fine tune" chromosome segregation. While there is no data to necessarily refute this assumption, this reviewer believes that it is not currently supported by experimental data. What is much more likely is that mis-segregation rates occur somewhat independently and aneuploid cells are under constant selection *in vivo* which may ultimately favor cells that have a given missegregation rate to become more viable under certain conditions.

I have read this submission. I believe that I have an appropriate level of expertise to confirm that it is of an acceptable scientific standard.

# Peer review: Referee response for version 1

**Published:** 2013-12-30

**Authors:** Jan Oxholm Gordeladze

**Affiliations:** 1 Institute of Basic Medical Sciences, Faculty of Medicine, University of Oslo, Oslo, Norway

The authors emphasize in their introduction to the present paper that: “ *The theoretical frame to understand such self-regulation resides with quasispecies theory*”, however, they introduce a change in its definition or the meaning of its objects. In particular, they propose a novel definition of the chromosomal master sequence. Finally, they discuss the potential origin of this self-regulatory ability in the wider context of developmental and comparative biology with emphasis on a putative “diversification factor”, defined as a cellular mechanism regulating the adaptation of aneuploidy rates, which is active in all embryonic and adult cells, as well as cancer stem cells. Furthermore, they refer to literature asserting that single clone cancer cells derived from an aneuploid cancer subpopulation, are capable of adapting their aneuploidy rate, and they display distinct aneuploid rates in distinct environments, namely primary and metastatic microenvironments.

 

This literature survey indicates that embryonic and adult stem cells display finely tuned, and distinct, aneuploidy rates, which are unrelated to the replication rates of the stem cells in question. Since many types of cancer cells partially inherit the hierarchical structure of the tissues from which they emanate, and are hypothesized to develop from stem like tumor cells, it seems highly probable that increased aneuploidy rates are used as an advantage to a given cell population, e.g. to increase the adaptability of stem or fast-dividing progenitor cells.

 

In this article the authors introduce the concept of a “diversification factor”, which is probably the key issue of the article. This factor may as well be extended to a cluster of “impacting signals”, which may fit the modern definition of epigenetics, encompassing signals defined as “epigenators”, “initiators” and “maintainers”, of which transcription factors (TFs), microRNAs and histone modifying enzymes (like histone deacetylases = HDACS) are known to play important parts, since they constitute a strongly interwoven network with feed-forward- and feed-back regulatory loops.

 

This network is under the influence of environmental/positional “factors” (epigenators, e.g. surface molecules, mechanical stimulation, environmental chemicals and others) recognized by the “initiators/maintainers” (e.g. TFs and microRNAs), and most importantly, this regulatory web is common to embryonic cells, stem cells, cancer stem cells, and adult (i.e. differentiated) cells. It is therefore highly probable that the degree of aneuploidy of cancer cells is highly dependent on environmental phenomena, and not solely on intrinsic or “inherited” traits localized within the cancerous cells themselves.

I have read this submission. I believe that I have an appropriate level of expertise to confirm that it is of an acceptable scientific standard.
